# Does intangible assets affect the financial performance and policy of commercial banks’ in the emerging market?

**DOI:** 10.1371/journal.pone.0272018

**Published:** 2022-08-25

**Authors:** Bayelign Abebe Zelalem, Ayalew Ali Abebe

**Affiliations:** Senior Lecturer, College of Business and Economics, Mizan-Tepi University, Mizan-Teferi, Ethiopia; Universiti Malaysia Sabah, MALAYSIA

## Abstract

In a digital and knowledge based economy, intangible assets are predominant and their role along with age and knowledge has become key success factors for firms. However, a very little attention was given to the intangible assets in the banking sectors’ in Ethiopia and the effect still not studied yet. Therefore, the aim of this study is to empirically examine the effect of intangible assets on the financial performance and policy of 17 commercial banks in Ethiopia from the year 2017 to 2020. Return on asset and equity were used to measure the financial performance and debt as a measure of financial policy. The intangible asset is used as the main explanatory variable and asset size and liquidity as control variables. Random effect estimation technique for panel data was used. The result revealed that intangible asset has positive effect on the financial performance measured both by ROA and ROE at 5% significance level while, negative effect on the financial policy of commercial banks in Ethiopia at 1% significance level. Moreover, the study found asset size has significant and positive effect on ROA and ROE at 1% and 5% significance level respectively. Liquidity ratio has also significant positive effect on the financial performance measured both by ROA and ROE at 5% significance level. Finally, the finding revealed asset size and liquidity ratio has significant positive effect on the financial policy of commercial banks in Ethiopia at 10% and 1% significance level respectively. Therefore, the study concludes that financial performance and policy is achieved not only by using physical assets but also using intangible assets. Thus, the boards and mangers of commercial banks’ ought to plan and maintain the appropriate ratio of intangible assets to total assets for securing sustainable development in achieving the maximization of shareholders wealth and to have optimum debt.

## 1. Introduction

Researchers and practitioners have reached a consensus that intangible assets play a vital role in the success and survival of firms in today’s economy [1]. In recent decades, the focus has shifted from the traditional financial statements that focus on tangible assets into intangible assets like innovation, knowledge, intellectual property, and goodwill. Intangible assets are assets that do not have a specific physical form, such as a company’s reputation, culture and value, brand name, technology, etc., but can make a significant contribution to creating business value [2]. As a result of recent mergers and acquisitions, companies have acquired different countries company at a higher price than total tangible assets of the companies. An important question that should be addressed is: Where does the source of a firm’s intrinsic value come from? It is due to intangible assets. These assets are one of the key sources of comparative advantages for companies [3]. These assets widen the market value and increase the profit of a company [4, 5].

Social, technical, economic and political developments have made notable changes in the work environment of various types of businesses. A consistent marked increase in intangible assets has been witnessed. Intangible assets lack physical presence, and their potential benefits are uncertain [6]. Appropriate intangible assets, which are considered as the roots of company value creation, help a company to achieve success [7]. Moreover, intangible assets are the major drivers of company growth and value in most economic sectors [8]. Stewart [9, 10] also concluded that intangible assets play an important role in determining company success. Similarly [11], stated that intangible assets played an important role in the success or failure of companies during the international financial crisis from 2007 to 2008 and found that companies can benefit from intangible assets only after a couple of years.

[7] Discovered that intangible assets have a positive relationship with the performance of a company. Several measures can be used to measure firm value, book value, market value, capitalized value and deductive application of human judgment as well as net worth adjusted for intangibles and idiosyncrasies [12].

Among the different activities that a firm may use to produce innovation, investment in intangibles occupies a top role. To produce innovation, investment in research and development and intellectual capital (human capital) is necessary. Likewise, advertising investment is critical for commercializing innovative product. These soft assets bring the competitive advantage and constitute the foundation for subsidiary expansion and increased performance for the firm [13]. It is well accepted in the literature that like traditional investment, investment in intangible assets has positive consequences for various performance results, including a firm’s market position, financial position, and firm value in the stock market [14]. Therefore, it is necessary to introduce a paradigm to measure them and establish a link between intangible assets and firm performance.

In spite of the impressive body of work related to intangible assets and firm performance, there remain several gaps on the relationship between intangible assets and firm performance. After the discussion of a vast literature, it is also not clear that how a firm’s performance dimensions are influenced by the interface of intangible assets as previous literatures in other countries do not make this relationship clear cut. Though, most of the researchers find a positive impact of intangible assets on firm performance [15–19]. However, the positive relationship between intangible asset and performance are often challenged by their counterparts who come up with negative or no impact [20–23]. Therefore, the strategic role of intangible assets, valuation approach of intangible assets, and the evidence of a positive relationship between intangible assets and firm performance are not widely accepted to the scholars, entrepreneur, and practitioners. After reviewing literature, it is also found that a large number of the methodologies have been connected utilizing the data samples in a variety of international settings including USA, Canada, UK, Australia, Germany, and Turkey raise the issue of generalization on the empirical results. Moreover, there is no investigation carried out yet on the effect of intangible assets on the financial performance and policy of commercial banks in Ethiopia. Based on the above mentioned gaps the current study conducted on the effect of intangible assets with the following objectives in the emerging market specifically in Ethiopia;

1. To investigate the effect of intangible assets on the financial performance of commercial banks in Ethiopia.
2. To examine the effect of intangible assets on the financial policy of commercial banks in Ethiopia.

The contribution of this study is that, first, the study is pivotal to managers in designing and exploiting relevant elements of intangible assets. Second, the study can be used as a guideline for boards when they plan to manage their investment in intangible assets. Furthermore, the study gives a direction to the board of the banks to plan and maintain the appropriate ratio of intangible assets to total assets for securing sustainable development.

The remainder of this paper is organized as follows. Section 2 presents the literature review and develops the hypotheses; Section 3 describes the research method; Section 4 reports result and discussions; the last section concludes the paper and offers suggestions and directions for future research.

## 2. Literature review and hypothesis development

### 2.1 Intangible assets

Studies on intangible assets have been conducted for decades. Researchers, such as [7, 8] have analyzed this topic from various perspectives [24]. Also studied on intangible assets and stated that no organizations or markets can purchase or sell intangible assets. Moreover, intangible assets are far from a homogenous category of assets [25]. According to [26], intangible assets are non-physical factors that contribute to, or are used in; the production of goods or the provision of services or that is expected to generate future productive benefits to the individuals or firms that control their use. Intangible assets are characterized by high risks, high uncertainty, firm-specificity, the absence of rivalry between uses and human capital intensity. Such characteristics, as well as the no tradability of most intangible assets, distinguish them from other types of assets [8].

Several approaches can be used to calculate intangible assets [27]. Listed a few main approaches, namely, based on market values, based on direct evaluation, based on income and based on scores [9]. As cited by [28] developed the calculated intangible value method to evaluate intangible assets, which is basically dependent on excess income. The basic logic of this method is that an investment in physical capital can only yield the average return prevailing in the industry; anything that exceeds the average yield is explained by the application of intellectual capital.

### 2.2 Intangible assets and financial performance

The effects of intangible assets and financial performance are widely revealed in the literature. Cost reduction is one of the important themes in traditional accounting, which may lead to a focus on strategies and an increase of a company’s value by improving customers’ equity leadership [29].

[30] Examined the influence of intangible assets (R&D expenditure) on the financial performance of listed information technology companies in Hong Kong by using ROA as a financial measure of the firms. The study found out that R&D investment and sales training are beneficial to the firms’ financial performance [24]. Examined the relationship between market value, dividend policy, solvency ratio, intangible value and company performance in Indonesia during the financial crisis of from 2006 to 2011. The results showed a significant relationship between the amount of intangible assets and the market value of a company.

[31] Studied on the way to achieving carbon neutrality in China. The main purpose of the article is to analyze the strategic decisions of the Chinese government under the influence of the environmental factor. The article analyzes the approaches to substantiating the ideology of environmental reforms (building an ecological civilization) and its key components, namely: the national climate program, the ecological red line, combating desertification and mitigating climate change, combating water pollution and eliminating water shortages. The study concluded that China has rich and interesting experience in solving the problem of modernizing production, ensuring its rational distribution and reducing the environmental burden on the territory, primarily in the framework of combating environmental pollution and climate change.

[18] Empirically examined the relationship between intangible assets, financial policies and financial performance and the firm value of companies going public in Indonesia. The study concluded that intangible assets have a positive and significant effect on financial performance and firm value [32]. Investigated the contribution of intangible assets to value creation and the financial performance of firms in German public limited companies. The findings showed that intangible assets contribute positively to the profitability and productivity of the firms.

[33] Studied on specific features of renewable energy development in the world and Russia. The purpose of the article is to study the theoretical and practical aspects of investment activities in the field of renewable energy in the world and in Russia. A systematic analysis of existing approaches to the assessment of financing mechanisms for renewable energy projects was carried out. The study examines the development of renewable energy, its benefits, and investments in the industry. The financial risks and barriers associated with financing renewable energy projects are considered. The development of the industry over the past 10 years is analyzed, taking into account the impact of the COVID-19 pandemic on the electricity industry in general and on renewable energy in a number of countries. It is established that the world is shifting to the use of renewable energy sources, and in Russia they are not being given due attention. It is revealed that the existing thermal generation units in Russia are of great age and are to be decommissioned in the near future. Moreover, the technical potential of wind and solar power plants in the Russian Federation is considered within the aim of diversifying electricity production.

[34] Investigated on the assessment of regional growth of small business in Russia with the objective of estimating small business development across regions of the Far Eastern District in Russia with regard to economic, social and environmental dimensions of sustainability using mathematical model. The results suggest that small businesses in the Far Eastern District will not be able to enhance their profitability and offer larger salaries by 2024.

[35] Investigated Legislative Regulation Financial Statement Preparation by Micro Entities using International Experience. The study analyzes the experience of legislative regulation of financial statement preparation by micro entities in the Commonwealth of Independent States, the European Union, and the United Kingdom. The legislative acts of the countries, national accounting standards and financial reporting standards serve as the methodological basis for the analysis. The study found that micro entities account for 96 percent of the small and medium-sized business sector, whose share in the gross domestic product was about 20.6% in 2019 according to Federal State Statistics Service estimates and which created about 33% of jobs. For comparison, in the OECD countries small and medium-sized businesses generate, on average, about 55% of GDP and about 59.1% of jobs, whereas in the EU countries the percentage is higher: 57.5% of GDP, and 65% of the employed.

[36] Studied on the relation of GDP per capita and corruption index. It is shown that corruption is best explained by GDP per capita and all other major macroeconomic indicators cannot add any statistically significant improvement to the models’ accuracy. Moreover, the growth of wealth in a society makes corruption recede and not the other way around (reducing corruption helps increase GDP per capita). However, the most counterintuitive finding of this research is the fact, that GDP per capita, adjusted by purchasing power parity, produces the model of a worse quality then just using plain GDP per capita.

[37] Examine the effect of an intangible asset on financial Performance of Banks in Nigeria using simple linear regression analysis. The result shows that intangible assets have a positive and significant effect on banks’ financial performances [38]. Suggest the better investment in intangible resources improve the financial performance of companies. Investment in human capital and technology has the perspective to improve the profitability and lack of investment can results in weaker firm performance [1]. Examine the association among investment in intangible assets and banking profitability in the short and long run using panel data. Their results indicate that intangible assets improve the banking performance in the long run.

[39] Researched on the effect of intangible assets on firms’ economic performance in listed telecommunication firms in China and found positive relationship. Intangible assets are the companies’ aggressive advantage and challenging to imitate. In the Chinese accounting standards, intangible asset include patents, copyright, franchise, and land-use right. There is an assumption that if in China, intangible asset owned with the aid of businesses can promote corporation’s performance higher than tangible assets, the market structure is aggressive and mature. Hence a positive and significant correlation was found between intangible assets and the firm’s profitability [40]. Conducted an exploratory data analysis on currently unrecorded internal intangible firm values. The results showed that the measure of the internally generated intangible assets affects firm value. Therefore, the study formulated the hypothesis as:

**H1:** Intangible assets have no effect on the financial performance of commercial banks’ in Ethiopia

### 2.3 Intangible assets and financial policy

Investments in intangible assets have an effect on debt policy and dividend policy within the company. Agency theory [41] argues that monetary policy is determined by the agency cost. Based on the unique characteristics of intangible assets, the agency cost is estimated to be higher in companies with intensive intangible assets. Intangible assets will increase the agency cost to shareholders because of more information and hidden action, also on debt holder agency cost asset substitution and underinvestment problem. Thus, investment in intangible assets will affect the company’s financial policy.

The companies that have high intangible assets will affect the company’s debt policy. Owners of companies that have high intangible assets can control the agency cost of debt by limiting the amount of risky debt. Agency cost of debt is increasing the cost of debt that occurs when there is a conflict of interest between managers and debt holder, where managers were more concerned with shareholders than debt holder. Agency cost of debt will be higher in companies that invest more in intangible assets. Companies that invest more intangible assets will possess a lower level of debt compared to companies that invest more in tangible assets [42]. Agency theory underline the relationship between intangible assets with debt policy consistent with the facts revealed by [43] that the R&D intensive in a company associated with the smaller debt in the company’s capital structure.

[18, 44] shed light on the effect of intangible assets on financial policy within a company and argue that intangible assets have effect on the company’s financial policy positively. Two theoretical arguments with differing opinions on the dividend policy are mentioned in the literature. One school of thought follows the opinion of [45] who argued that dividends should have no impact on firm value. By contrast [46], considered dividend policy as relevant and exerts influence on firm value [47]. Concluded that information obtained from financial statements is relevant for investors in decision making and can explain the size of the stock market. Thus, ratios derived from financial statements have a significant relationship with stock market indicators. In contrast to the aforementioned views [44], examined the impact of the level and the type of intangible assets on six major financial and governance policies by using two UK cross-sectional samples. The results showed that intangible assets have a significant negative impact on debt and dividend payout.

Moreover, as per signaling argument [48] companies with high intangible assets, must pay high dividends to provide a good quality signal to investors. In terms of dividend policy, the theory is in contrast with the pecking order theory and agency theory. Thus, in line with this theory, companies with high R&D tend to pay lower dividends [49]. In the same way the study formulated the hypothesis as:

**H2:** Intangible assets have no effect on the financial policy of commercial banks’ in Ethiopia

**Fig 1:**
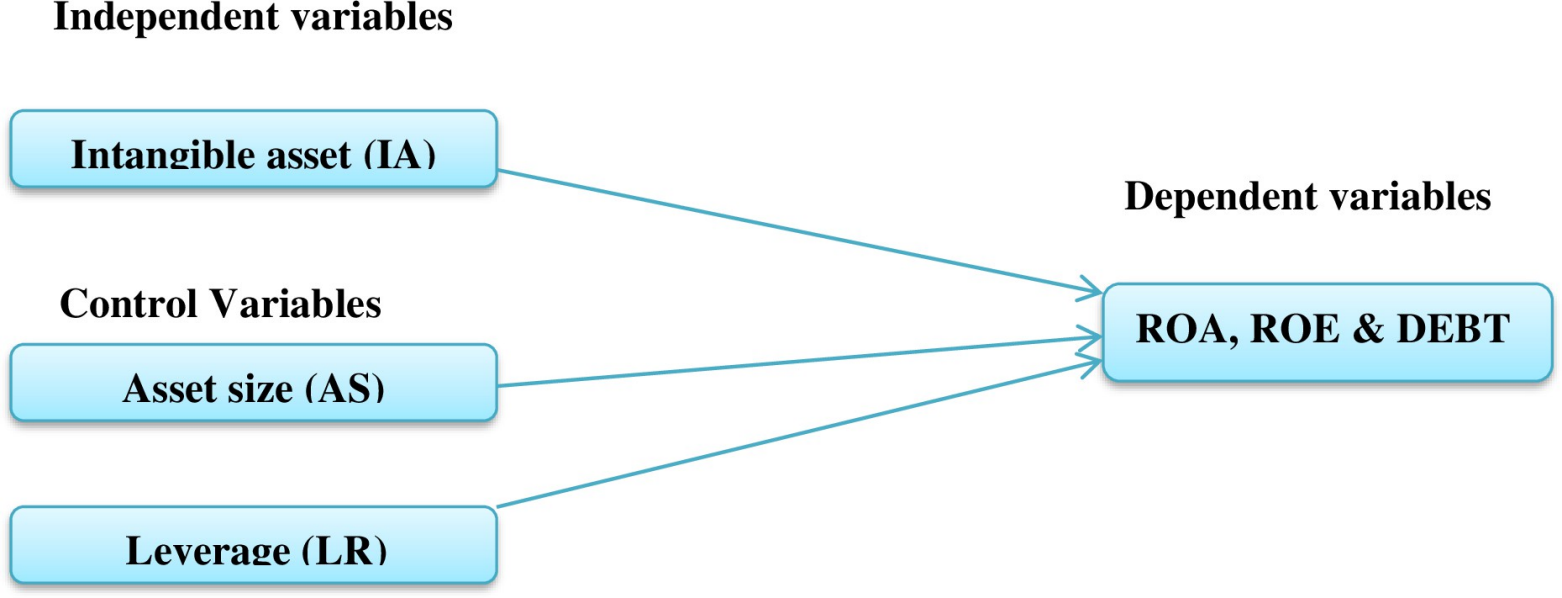
Conceptual framework. Sources: Own design 2021.

Fig 1 below shows the relationship between dependent and independent variables of the study. Return on asset (ROA) is the dependent variable that measures commercial banks’ profitability in relation to its total assets. Return on equity (ROE) is also the dependent variable that measures the ability of commercial banks’ to earn a return on its equity investments. Debt (DEBT) is the third dependent variable used to measure the financial policy of commercial banks’. Intangible asset (IA) is the major independent variable that has not physical in nature. Asset size (AS) is the other independent variable that generally used to capture commercial banks’ potential economies or diseconomies of scale. The last independent variable is liquidity ratio (LR) that is used to assess commercial banks’ capacity to meet its short term loan obligations.

## 3. Research method

The study is empirical, descriptive, and relational. According to time reference of research it is longitudinal (2017–2020), and research philosophy is inductive. Sources of data are secondary, published audited annual report. The study uses quantitative research as a method for data collection and analysis. The unit of analysis consists of all 17 (seventeen) commercial banks in Ethiopia that have four years data.

### 3.1 Model specifications

The information that the researcher utilized in this investigation were board information. Board information includes the pooling of perceptions on a traverse a few timespans and gives results that are just not distinguishable in unadulterated cross-areas or unadulterated time-arrangement considers [50]. The overall type of the board information model can be determined mathematically as:

Yit=α+βxi,t+∑i,t

In this condition Yi,t speaks to the reliant variable, which is the commercial banks financial performance for model one and model two respectively. Moreover, Yi,t represents again the financial policy of commercial banks for model three and Xit contains the arrangement of logical factors in the model. The addendums i and t signifies the cross sectional and time arrangement measurement separately. Likewise α taken to be consistent after some time t and explicit to the individual cross sectional unit i. In light of the above model and on the base of chosen factors the current investigation utilized econometric model demonstrated as follows.

### Model 1:


ROA=f(IA,AS,LR) 1

ROAi,t=α+β1IAi,t+β2ASi,t+β3LRi,t+∑i,t

Where:

ROA = Return on asset, IA = Intangible assets, AS = Asset size, LR = Liquidity ratio, and ∑ = Error term

### Model 2:


ROE=f(IA,AS,LR) 2

ROEi,t=α+β1IAi,t+β2ASi,t+β3LRi,t+∑i,t

Where:

ROE = Return on equity, IA = Intangible assets, AS = Asset size, LR = Liquidity ratio and ∑ = Error term

### Model 3:


DEBT=f(IA,AS,LR) 3

DEBTi,t=α+β1IAi,t+β2ASi,t+β3LRi,t+∑i,t

Where:

DEBT = Debt, IA = Intangible assets, AS = Asset size, LR = Liquidity ratio and ∑ = Error term

### 3.2 Variables construction

**3.2.1 Dependent variables.** Return on Asset (ROA), Return on Equity (ROE) and Debt (DEBT) of bank i at time t is used as dependent variables in this study respectively.

**3.2.2 Independent variables.** As the major independent variable, the study used intangible assets and asset size and liquidity as control variables for bank i at time t, denoted as IAi,t, ASi,t, and LRi,t respectively. The study variables employed in the empirical analysis of this study are summarized in Table 1 below.

**Table 1:** Variables and definitions.

Variables	Symbol	Definitions	Expected direction of effect
**Dependent variables**			
**Return on Asset**	ROA	The ratio of net income after tax to total asset of bank i at time t	
**Return on Equity**	ROE	The ratio of net income after tax to total equity of bank i at time t	
**Debt**	DEBT	The ratio of debt to equity of bank i at time t	
**Independent variables**			
**Intangible assets**	IA	The ratio of value given to intangible assets to total assets of bank i at time t	**+**
**Asset size**	AS	The annual logarithm of the total asset of bank i at time t	**+/-**
**Liquidity ratio**	LR	The ratio of current asset to current liability for bank i at time t	**+**

Source: Own design 2021

## 4. Result and discussion

### 4.1 Descriptive statistics

Table 2 below indicated that the mean value of return on asset of commercial banks in Ethiopia was 2.61064percent with standard deviation of 3.55684percent. The minimum and maximum value of return on asset that banks earned was -0.01167 and 29.7percent respectively. The mean value of return on equity for commercial banks in Ethiopia was 16.78312percent with standard deviation of 7.81595percent. The minimum and maximum value of return on equity for commercial banks in Ethiopia was -0.107 and 45.628 percent respectively. The average debt of commercial banks in Ethiopia under the period of the study was 6.328005 with standard deviation of 2.979594. The minimum and maximum debt of commercial banks was 0.148 and 15.444 respectively. The mean value of intangible assets was 0.23574percent with standard deviation of 0.42677percent. The minimum and maximum value of intangible assets was 0.00123 and 3.138percent respectively. The average value of asset that commercial banks hold was 7.682408 with standard deviation of 1.260531. The minimum and maximum assets of the bank under the period of the study were 6.315 and 11.9134 respectively. Finally, the table shows that the average value of liquidity was 1.105583 with standard deviation of 0.3533913. The minimum and maximum liquidity of banks under the period of the study were 6.315 and 11.9134 respectively.

**Table 2:** Descriptive statistics of the variables.

Variables	Obs	Mean	Std. Dev.	Min	Max
**ROA**	68	.0261064	.0355684	-.0001167	.297
**ROE**	68	.1678312	.0781595	-.00107	.45628
**DEBT**	68	6.328005	2.979594	.148	15.444
**IA**	68	.0023574	.0042677	.0000123	.03138
**AS**	68	7.682408	1.260531	6.315	11.9134
**LR**	68	1.105583	.3533913	.193	3.2112

Source: Stata 14 Summery of statistics result

### 4.2 Trend analysis of intangible assets

**Fig 2:**
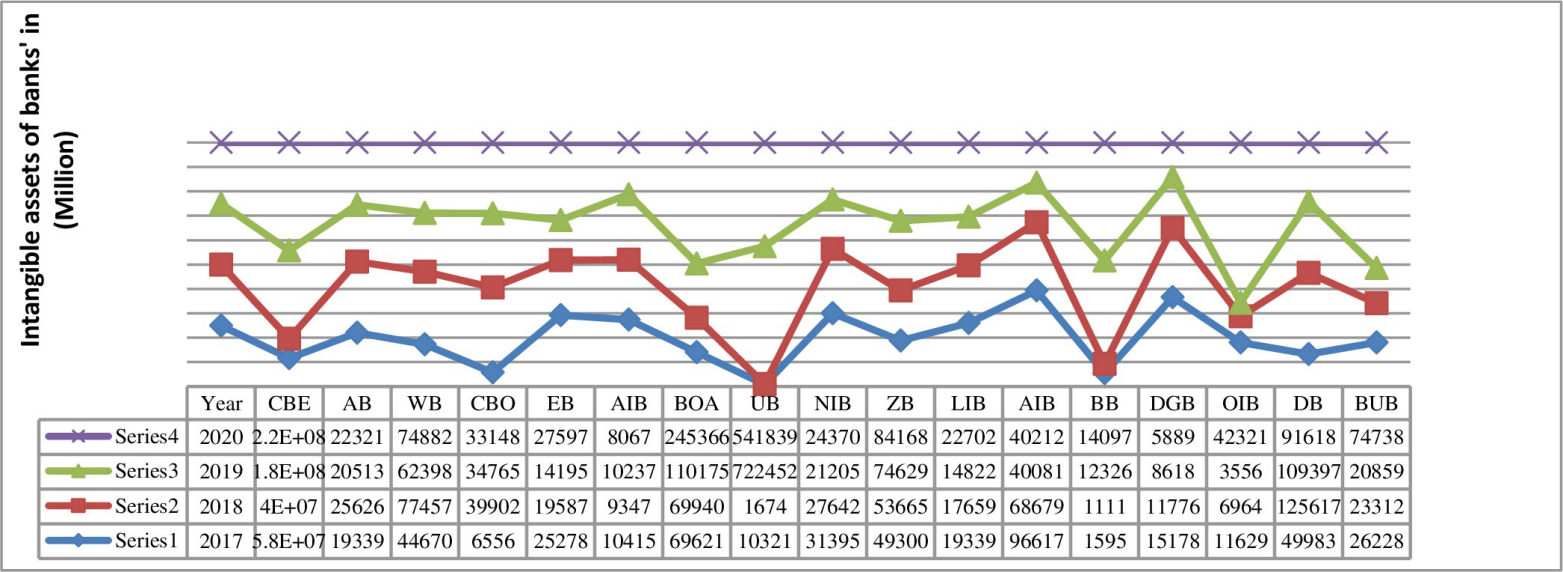
Trend of intangible assets of seventeen (17) commercial banks’ in Ethiopia. Source: Own computation 2021.

The report of trend analysis will describe how the total amount of intangible assets behaves over time. Do they follow increasing, decreasing, or staying the same? To find out the answer, the study analyzes and discusses the trend. Fig 2 below shows the trend of intangible assets of seventeen commercial banks for the years from 2017 to 2020. It is pretty much clear from the line chart that there has been a large increase in the total amount of intangible assets for commercial bank of Ethiopia (CBE) from 2017 to 2020 respectively. The trend also showed that the intangible assets for Abay bank (AB), Wogagen bank (WB), Cooperative bank of Oromia (CBO), Enat bank (EB), Awash international bank (AIB), bank of Abyssinia (BOA), Nib international bank (NIB), Zemen bank (ZB), Lion international bank (LIB), awash international bank (AIB), Addis international bank (AIB), Debub global bank (DGB), and Dashen bank (DB) increased from 2017 to 2020 respectively. While, the intangible assets for united bank (UB) 2018 is less than the intangible assets of 2017 and the intangible assets of 2020 is less than the intangible assets of 2019. Similarly, the intangible assets of Berhan bank (BB), 2018 is less than 2017 intangible assets and the intangible assets increased for 2019 and 2020 respectively. The intangible assets of Oromia international bank (OIB) decreased from 2017 to 2019 respectively and increased in 2020. Finally, the trend showed that the intangible asset of Buna bank (BUB) decreased from 2017 to 2019 and increased in 2020.

### 4.3 Correlation matrix

Table 3 below shows there is positive correlation between intangible assets, return on asset and equity with (r = 0.0335). However, there is a negative correlation between intangible assets and debt of banks with (r = -0.1332). The correlation result also shows there is a negative correlation between asset size, return on asset, return on equity and debt with (r = -0.1465). Finally the correlation result indicates that liquidity ratio of commercial banks was positively correlated with return on asset, return on equity and debt of commercial banks in Ethiopia with (r = 0.0410).

**Table 3:** Correlation matrixes.

ROA	ROE	DEBT	IA	AS	LR
**ROA**	1.0000				
**ROE**	0.0335				
**DEBT**	-0.1332	-0.1006	1.0000		
**IA**	0.0839	0.0895	-0.1837	1.0000	
**AS**	-0.1465	0.0604	0.5110	-0.2031	1.0000
**LR**	0.0410	-0.0170	0.0196	-0.0906	0.1881 1.0000

Source: Stata 14 Correlation analysis

### 4.4 Random effect (REM) and fixed effect model (FEM)

There are broadly two classes of panel estimator approaches that can be employed in financial research these are fixed effects models (FEM) and random effects models (REM) [51]. To check which of the two (FEM or REM) models provide consistent estimates for this study; Hausman test was employed and the following hypothesis was developed;

**Ho**. Random effect model is appropriate for the study
**H1**. Random effect model is not appropriate for the study

The null hypothesis of the test was that the random effect method is the preferred regression method. The p-value for the test in Table 4 below is (47), (75.7) and (85.66) percent for model 1, 2 and 3 respectively which indicate that the null hypothesis was not rejected. Accordingly, random effect model (REM) was employed to estimate the relationship between the dependent and the independent variables.

**Table 4:** Random and fixed effect model.

Model 1	Fe	Re	Difference	S.E.
**IA**	-.091117	.4408043	-.5319213	.8289742
**AS**	.0151723	-.0038849	.0190572	.0134137
**LR**	.0020857	.0068544	-.0047687	.0062314
Prob>chi2 = 0.47				
Model 2	Fe	Re	Difference	S.E.
**IA**	.2647825	.6602381	-.3954556	.5759087
**AS**	.0319677	.0185021	.0134656	.0144398
**LR**	.0854471	.0859853	-.0005382	.0047047
Prob>chi2 = 0.757			
Model 3	Fe	Re	Difference	S.E.
**IA**	-1.180908	26.72804	-27.90895	37.11176
**AS**	.6047241	.1998026	.4049214	.7468073
**LR**	-.175389	-.1513459	-.0240431	.2894511
Prob>chi2 = 0.8566				

Source: Stata 14 hausman test

### 4.5 Regression results and discussions

The adjusted *R*^2^ value below in Tables 5–7 indicates that 53.421, 64.742 and 51.62 percent of the total variability of return on asset, equity and debt of commercial banks was explained by the variables in the models respectively.

**Table 5:** Regression result -REM of Model 1.

DEBT	Coef.	Std. Err.	z P>|z|	[95% Conf. Interval]
**IA**	.6379981	72.35952	-0.88 0.0378**	-205.6219 78.02225
**AS**	.9272482	.3506781	2.64 0.008***	.2399317 1.614565
**LR**	.4787315	.7976296	0.60 0.0541**	-1.084594 2.042057
**Cons**	-1.174369	2.775904	-0.42 0.672	-6.615041 4.266302
Adjusted R^2^ = 51.62				

Source: Computed from stata 14. Note.

** and * indicates that significant at 1 and 5persent

**Table 6:** Regression result -REM of Model 2.

ROA	Coef.	Std. Err.	z P>|z|	[95% Conf. Interval]
**IA**	.4408043	1.102771	0.40 0.0423**	-1.720587 2.602196
**AS**	.0038849	.0040189	-0.97 0.0334**	-.0117618 .0039919
**LR**	.0068544	.0129885	0.53 0.0298**	-.0186026 .0323114
**Cons**	.0473347	.0327759	1.44 0.149	-.0169049 .1115743
Adjusted R^2^ = 53.421				

Source: Computed from stata 14. Note.

** indicates that significant at 5percent.

**Table 7:** Regression result -REM Model 3.

ROE	Coef.	Std. Err.	z P>|z|	[95% Conf. Interval]
**IA**	-.6602381	1.678105	0.39 0.00694***	-2.628786 3.949263
**AS**	.0185021	.0107598	1.72 0.086*	-.0025866 .0395909
**LR**	.0859853	.0179188	4.80 0.000***	.0508651 .1211054
**cons**	-.0709297	.0842544	-0.84 0.400	-.2360653 .0942058
Adjusted R^2^ = 64.742				

Source: Computed from stata 14. Note.

*** and * indicates that significant at 1 and 10persent

#### 4.5.1 Intangible assets, asset size, liquidity and return on asset (ROA) model 1

The **REM** of Table 5 below shows the coefficients of key variable intangible assets along with other explanatory variables are statistically significant at five percent level of significance. The coefficient value of intangible assets is 0.4408034 meaning a one percent increase in intangible assets leads to increase in ROA by 44.08034 percent. This interpretation indicates that intangible assets positively influence the banks’ financial performance. The study finding is consistent with the study finding of [39] that used recent data on 17 Chinese publicly listed firms during 2014–2016 to examine the relationship between intangible assets and firm performance [52, 53]. also observed a positive coefficient value of intangible assets in their regression model in the context of European and Bangladesh economy, respectively. Moreover, the finding of this study is consistent with [40, 54, 55], proving that the intangible assets has positive and significant impact on company performance as represented by the ROA.

The first control variable of model one is asset size which is positively associated with the banks’ financial performance, measured by ROA. Larger banks are supposed to perform better. Consistent with the prior findings, the positive coefficient value of firm size suggests that sample firms are large enough to exploit the economies of scale as well as have better bargaining power over their competitors and suppliers. The coefficient value of firm size is 0.0038849 meaning an increase of five percent intangible assets leads to increase in ROA by 0.38849 percent. The finding of this study is consistent with [56] established that firm size positively influences financial performance of a firm. The second control variable of model one is liquidity which is positively associated with the banks financial performance, measured by ROA with the coefficient of .0068544 at five percent significance level. Meaning taking other explanatory variables in the model constant a one percent increase in liquidity ratio increased the financial performance of commercial banks measured by ROA to 0.68544percent. The finding of this study is consistent with [57] that proves the higher the bank’s current asset capabilities to meet its’ current liabilities and the current ratio has a positive and significant effect that the current ratio can be used to predict stock returns.

#### 4.5.2 Intangible assets, asset size, liquidity and return on equity (ROE) model 2

As the **REM** indicates below in Table 6 intangible assets are statistically significant at five percent level of significance. The coefficient of intangible assets is 0.6602381 meaning other thing remains constant a one percent increase in intangible assets leads to 66.02381 percent increase in financial performance measured by ROE. The study finding is consistent with [58–60] that they concluded firm performance can be improved operationally and strategically by using intangible assets.

Similar, to model one model two of the regression result used asset size as control variable which is positively associated with the banks’ financial performance measured by ROE. The coefficient of asset size is 0.0185021 meaning one percent increase in asset size leads the bank to earn 1.85021 returns from their equity. The finding of the study is consistent with the finding of [61–65] that they report a positive association between bank size and profitability, which they interpret as evidence in support of the market power efficiency theory. However, the study is in contrary with [66] that suggests bank size had negative impact on the profitability of commercial banks, which supports the idea that economies of scale and synergies arise up to a certain level of size beyond that level, financial organizations become too complex to manage and dis economies of scale arise. The effect of size could therefore be nonlinear; meaning profitability is likely to increase up to a certain level by achieving economies of scale and decline from a certain level in which banks’ become too complex and bureaucrat.

The other control variable used in this study is liquidity that is found to be statistically significant and positively correlated with profitability measured by ROE at one percent significance level and coefficient of 0.0859853 meaning a one percent increase in liquidity leads 8.59853 percent increase in financial performance measured by ROE. The finding of this study is consistent with [64, 65, 67] that support the idea of classical theory of interest that suggests the higher the liquidity the greater the profitability. However, the result of this study is in contrary with [68] that they suggest liquidity had negative impact on the profitability of commercial banks because productive asset become idle.

#### 4.5.3 Intangible assets, asset size, liquidity and debt (DEBT) model 3

As shown in Table 7 below the coefficient of intangible assets of model 3 is negatively associated with DEBT with a coefficient of -0.6379981. The negative relationship between investment in intangible assets and DEBT meaning considering other explanatory variables constant a one percent increase in intangible assets leads to a decrease in DEBT by 63.79981percent. This finding is consistent with [44] that examined the level and type of intangible assets on six major financial and governance policies by using two UK cross-sectional samples. The results showed that intangible assets have a significant negative impact on debt.

This study used both asset size and liquidity of banks as control variables and found that positive and significant relationship with DEBT at five percent significance level with a coefficient of 0.9272482 and 0.4787315 meaning a one percent increase in asset size and liquidity leads to 92.72482 and 47.87315 percent increase in debt finance respectively. The finding is consistent with (Espinosa [69], that supports large banks can practice economies of scale, have better business awareness, and can recruit better managers, and large sizes can allow greater specialization meaning large banks can also calculate market power or the level of concentration in the industry by finding external sources of financing for their operations.

Regarding to liquidity the finding is consistent with [70] and static trade off theory which states greater liquidity would ensure that banks are able to fulfill their short term obligations. It can therefore be understood that the effect of liquidity has a greater impact on banks debt financing. Overall, the positive effect of liquidity on debt decisions indicates that commercial banks’ of Ethiopia see liquidity as a guarantee that they can survive and perform their duties when it is difficult for a banks’ to raise funds or in a lower earnings situation, or in periods of very high capital costs.

### 4.6 Classical linear regression model assumptions (CLRMA)

#### 4.6.1 Heteroscedasticity

The assumption of Homoscedasticity states that variance of the errors is constant. According to [71], given the value of X, the variance of ui is the same for all observations. If the errors do not have a constant variance, it is said that the assumption of homoscedasticity has been violated. This violation is termed as heteroscedasticity. In this study Breusch- Pagan test was used to test for existence of heteroscedasticity across the range of explanatory variables for model one, two and three respectively. Therefore, it is obtained from Breusch Pagan test in Table 8 below the p-value of 0.13, 0.21 and 0.27 respectively that heteroscedasticity is not the problem for this study.

**Table 8:** Breusch-Pagan Test.

Model 1	Model 2	Model 3
**chi2(1) = 10.32**	4.46	10.29
**Prob > chi2 = 0. 13**	0.21	0.27

Source: Stata 14-Het Test Result

#### 4.6.2 Multi collernerity

Multicollinearity is one of the problems in multiple regression analysis. It is usually regarded as a problem arising out of the violation of the assumption that explanatory variations are linearly independent [71]. However, the mire satisfaction of this assumption does not prevent the possibility of an approximate linear dependence among the explanatory variables. Table 9 below shows that there are no tolerance values below 0.1 and the values of VIF less than 10, suggesting model one, model two and model three indicates that multicollinearity is not a potential problem for this study.

**Table 9:** Test for multi-collernerity.

Variable	VIF	1/VIF
**AS**	1.08	0.929716
**IA**	1.05	0.955890
**LR**	1.04	0.961760
**Mean**	**1.05**	

Source: Stata 14 multi-collernerity test

#### 4.6.3 Normality

[51] Pointed out that in order to conduct hypothesis test about the model parameter, the normality assumption must be satisfied. The normality assumption is about the mean of the residuals is zero. Accordingly, the study used Shapiro- Wilk test for normal data. Based on this test if the p-value is less than 0.05, then the null hypothesis that the data are normally distributed is rejected. If the p-value is greater than 0.05, then the null hypothesis has not been rejected. Therefore, the Shapiro- Wilk test of the study in Table 10 below provided p-value of 0.08210, 0.0691 and 0.2154 for model one, two and three respectively that is greater than the p value of 0.05.

**Table 10:** Normality test: Shapiro.

Variable	Obs	W	V	Z	Prob>z
**Model (1)**	64	0.30257	39.928	7.977	0.08210
**Model (2)**	64	0.94239	3.298	2.582	0.0691
**Model (3)**	64	0.26974	41.807	8.076	0.2154

Source: Stata 14 swilk test

#### 4.6.4 Auto correlation test

This is an assumption that the errors are linearly independent of uncorrelated with one another. If the errors are correlated with one another, it would be stated that they are auto correlated. The Durbin-Watson statistic ranges in value from 0 to 4. A value near 2 indicates non-auto correlation; a value toward 0 indicates positive autocorrelation; a value toward 4 indicates negative autocorrelation. To check the problem of auto correlation, the study used Durbin Watson test. Table 11 below shows the DW test statistic value which is 1.96950, 1.98120 and 1.74104 respectively for model one, two and three respectively. The test statistic was clearly between the 1.5, which is in the inclusion area and 4 minus the upper limits and thus the null hypothesis of no evidence of autocorrelation was not rejected and no significant residual autocorrelation was presumed.

**Table 11:** Autocorrelation test: Durbin Watson.

	Durbin Watson test
**Model (1)**	statistic (4, 64) = 1.96950
**Model (2)**	statistic (4, 64) = 1.98120
**Model (3)**	statistic (4, 64) = 1.74104

Source: Stata 14 DW test

## 5. Conclusion

Intangible assets are the major drivers of company growth and value in most economic sectors. Appropriate intangible assets, which are considered as the roots of company value creation, help a company to achieve success. Moreover, intangible assets are one of the key sources of competitive advantage however, in the context of Ethiopia the commercial banks gave value below 1% for intangible assets meaning more emphasis is given for the tangible assets than intangible assets. Therefore, studies on the intangible assets is worth well.

The main objective of this study is to empirically examine the effect of intangible assets on the financial performance and policy of commercial banks operated in Ethiopia. The intangible assets are measured the values given to the intangible assets in the balance sheet of banks to total assets of the banks. The financial performance was measured using both ROA and ROE and the financial policy was measured using DEBT. Moreover, the study used asset size and liquidity as control variables.

The regressions result shows a positive and significant relationship between intangible assets and financial performance measured both by ROA and ROE however, significant and negative relationship with the financial policy of commercial banks measured by DEBR in Ethiopia. In addition, the study founded that both control variables asset size and liquidity are significant and positive effect on the financial performance and policy of commercial banks in Ethiopia. Therefore, the study concludes that better financial performance and policy is achieved not only by using physical assets but also by intangible assets. Moreover, optimum asset size and liquidity are also crucial for better banks financial performance and policy.

## 6. Policy implications

The findings of the study have some important theoretical as well as managerial implications. Theoretically the finding supports those banks’ financial performance and policy as a function of organizational resources. The study also extends the theoretical doctrine in terms of association among intangible resources and banks’ financial performance providing the data from Ethiopia. As for practitioner implications, the findings are pivotal to managers in designing and exploiting relevant elements of intangible assets. The findings can be used as a guideline for boards when they plan to manage their investment in intangible assets. The findings also give a direction to the board of the banks to plan and maintain the appropriate ratio of intangible assets to total assets for securing sustainable development. Stakeholders should recognize the importance of intangible assets and increase investment in soft assets this will result innovation in products and services and help the banks’ to fight in the global competitive markets.

Moreover, investors can use the proposed models to select their portfolios that have a track record for continuous investment in intangible assets in an efficient and sustainable way. Thus, the proposed models will make easy for the general investors to take prudent investment decisions in buying or selling share of intangible assets intensive banks’. In this way, the findings of the study will provide useful information to the internal and external users of financial statements of intangible assets intensive banks’. Finally, banks should optimize their asset size to get economies of scale and greater knowledge of markets. In addition, banks should optimize liquidity because banks with optimum liquidity have the capacity to fulfill their contractual obligation and, therefore, resort to funding through debt and optimum liquidity would ensure that banks’ will meet their short term duty and achieve the shareholders wealth.

The findings of the research are not without limitations. First, the proxy used to measure the financial performance and policy was limited to ROA, ROE and DEBT respectively. Therefore, further research may be carried out including other financial performance and policy measures. Second, the control variables used were limited to asset size and liquidity. Therefore, further research will be conducted using other explanatory variables that affect the financial performance and policy. Finally, the study finding focused on commercial banks other than other institutions. Therefore, investigations will be made including other institutions.

## Supporting information

S1 Data (XLSX)
